# Exploring the Causal Relationship Between Inflammatory Bowel Disease and Bell's Palsy Based on Inflammatory Proteins: A Mendelian Randomization Study

**DOI:** 10.1002/brb3.70715

**Published:** 2025-08-04

**Authors:** Daofeng Fan, Wenbao Wu, Jiaqian Dai, Yinjuan Chen, Qingqing Lian, Changbo Zhao, Binfu Que, Rui Qiu

**Affiliations:** ^1^ Department of Neurology Longyan First Hospital Affiliated to Fujian Medical University Longyan Fujian China; ^2^ Department of Acupuncture and Moxibustion Longyan First Hospital Affiliated to Fujian Medical University Longyan Fujian China; ^3^ Department of Acupuncture and Moxibustion Xingwen County Hospital of Traditional Chinese Medicine Yibin Sichuan China

**Keywords:** Bell's palsy, inflammatory bowel disease, inflammatory proteins, two‐sample Mendelian randomization

## Abstract

**Background:**

The relationship between inflammatory bowel disease (IBD) and Bell's palsy remains unsubstantiated. This study aims to investigate the causal relationship between IBD and Bell's palsy.

**Methods:**

Using the two‐sample Mendelian randomization (MR) method to explore the relationship between IBD and Bell's palsy. We applied Bell's palsy summary statistics from GWAS statistics for 91 inflammatory proteins and FinnGen R10 and IBD summary statistics from the International Inflammatory Bowel Disease Genetics Consortium.

**Results:**

The two‐sample MR study indicates a significant positive association between IBD and Bell's palsy (OR: 1.13, 95% CI [1.03–1.23], *p* = 0.0065) and between Crohn's disease and Bell's palsy (OR: 1.10, 95% CI [1.02–1.18], *p* = 0.0088). After applying Bonferroni correction, IBD remained significantly correlated with Bell's palsy. Subsequently, the causal relationship between circulating inflammatory proteins in Bell's palsy and inflammatory bowel disease samples was reevaluated. The results of the MR study on inflammatory proteins in these two diseases suggest that the C‐X‐C Motif Chemokine Ligand 5 (CXCL5) is a potential protective factor and interleukin_17C (IL_17C) is a risk factor for these two diseases. Conversely, Signaling Lymphocytic Activation Molecule Family Member 1 (SLAMF1) is a protective factor for Bell's palsy and a risk factor for inflammatory bowel disease.

**Conclusions:**

The findings indicate that IBD may be a risk factor for Bell's palsy at the genetic level. CXCL5, IL_17C, and SLAMF1 are possible co‐acting pathways between Bell's palsy and inflammatory bowel disease. These findings may provide new targets for the treatment of both diseases.

## Graphical abstract

Inflammatory bowel diseasemay be a risk factor for Bell's palsy at the genetic level. CXCL5, IL_17C, and SLAMF1 are possible co‐acting pathways between Bell's palsy and inflammatory bowel disease.

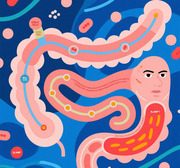

**Abbreviations**
CD40: cluster of differentiation 40
CXCL5: C‐X‐C motif chemokine ligand 5
FGF21: fibroblast growth factor 21
IL_17C: interleukin‐17C
IL_18: interleukin‐18
SLAMF1: signaling lymphocytic activation molecule family member 1
TGFα: transforming growth factor alpha

## 1 Introduction

Inflammatory bowel disease (IBD) includes a group of inflammatory disorders that affect the gastrointestinal tract, mainly Crohn's disease (CD) and ulcerative colitis (UC) (Gilliland et al. 2024). These conditions cause various symptoms linked to inflammation in the intestines, like abdominal pain, fever, vomiting, diarrhea, rectal bleeding, anemia, and weight loss (de Lange and Barrett 2015). The prevalence of IBD is higher in developed countries, with Europe and North America (Morshedzadeh et al. 2022). The highest incidence rates in Europe are observed, with UC affecting 505 per 100,000 people and CD affecting 322 per 100,000 people. In North America, the rates are 249 per 100,000 people for UC and 319 per 100,000 people for CD (Ashton and Beattie 2024). The number of IBD cases worldwide is increasing yearly, significantly burdening healthcare systems worldwide (Yadav et al. 2016). Although the exact etiology of IBD is not fully understood, its pathogenesis involves a complex interaction of genetic, immunologic, and environmental factors. In recent years, more and more studies have focused on the impact of IBD on general health, especially its potential association with various neurological disorders.

Bell's palsy (BP), also known as idiopathic facial paralysis, is characterized by paralysis of the facial muscles due to inflammation of the facial nerve (W. Zhang, Xu, et al. 2020; Heckmann et al. 2019). Most cases resolve within a month, but about 30% of patients recover within 6 months, and some may have incomplete recoveries, leading to ongoing facial weakness and spasms (Sandell 2004). BP can impact individuals of all ages, with an annual incidence ranging from 20 to 30 cases per 100,000 people in different populations (Atan et al. 2015; Luu et al. 2021). Facial spasms and exposure keratitis linked to BP can significantly affect patients’ quality of life, potentially impacting their work and productivity (Eviston et al. 2015). Visible facial asymmetry and limited facial movements can cause social issues, depression, and avoidance behaviors (Skuladottir et al. 2021). Previous studies have suggested that systemic inflammation and immune system abnormalities may play an important role in the pathogenesis of BP (W. Zhang, Xu, et al. 2020). As a result, more attention is being given to studying ways to prevent BP effectively.

Although IBD and BP differ in clinical manifestations and organs involved, there is growing evidence of a possible association between the two. Epidemiologic studies have shown that the prevalence of BP is higher in patients with IBD than in the general population, suggesting that the two diseases may share certain pathophysiologic mechanisms (Heckmann et al. 2019). However, since most of these studies were based on observational data, a causal relationship could not be established. Therefore, it has become particularly important to explore the causal relationship between IBD and BP using genetic epidemiology.

Mendelian randomization (MR) is a statistical method that employs single‐nucleotide polymorphisms (SNPs) as instrumental variables (IVs) to assess causal relationships, thereby mitigating confounding and reverse causation biases commonly encountered in traditional observational studies (Skrivankova et al. 2021; Davies et al. 2018). By analyzing the associations between genetic variants and both IBD and BP, we can provide a more accurate evaluation of the causal relationship between these two diseases. This study aims to utilize MR to assess the causal relationship between IBD and BP through genetic IVs, with a particular focus on the role of inflammatory proteins in this association.

## 2 Methods

### 2.1 Study Design

**FIGURE 1:**
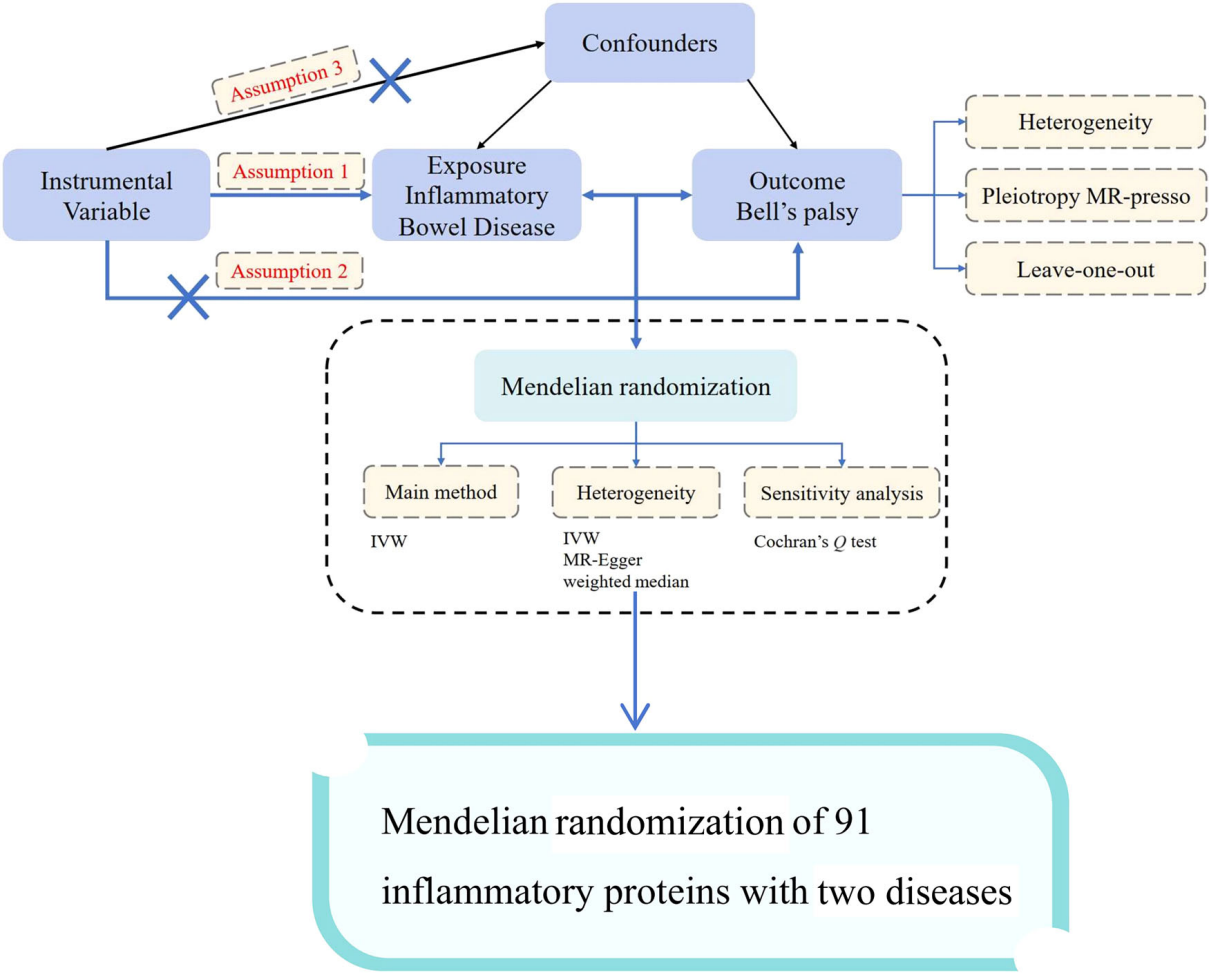
The study flowchart.

In this study, we used a two‐sample MR approach, a causal inference method based on genetic variation, to assess the causal relationship between IBD and BP. We utilized summary statistics data for IBD from the International Inflammatory Bowel Disease Genetics Consortium (IIBDGC) for IBD and from the FinnGen R10 for BP. The use of these datasets complied with appropriate patient consent and ethical approvals. The specific process is illustrated in Figure 1.

### 2.2 Data Sources

Summary‐level data for IBD, including CD and UC, were obtained from the IIBDGC consortium, including 12,882 IBD cases, 5956 CD cases, and 6968 UC cases (Liu et al. 2015). Additionally, summary‐level data for BP were sourced from the FinnGen Consortium (consisting of 3781 BP cases and 360,538 controls). GWAS statistics for 91 inflammatory proteins were summarized from 14,824 Europeans (Zhao et al. 2023). The sources and detailed information of these datasets are presented in Table 1.

**TABLE 1:** Data sources.

Data	Sample size (cases/controls)	Ancestry	Significance level	Data sources
Inflammatory bowel disease	12882/21770	European	5e−8	IIBDGC (ieu‐a‐31)
Crohn's disease	5956/14927	European	5e−8	IIBDGC (ieu‐a‐30)
Ulcerative colitis	6968/20464	European	5e−8	IIBDGC (ieu‐a‐32)
Bell's palsy	3781/360538	European	5e−8	FinnGen R10

### 2.3 IV Selection

SNPs, representing variations at a single nucleotide position in the DNA sequence, constitute the most prevalent form of genetic variation among humans. For SNPs to serve as IVs in MR studies, they must fulfill three critical criteria (Birney 2022; Carter et al. 2021). First, the SNP must exhibit an association with the exposure under investigation. The SNP should not be associated with confounders that might influence the relationship between the exposure and the outcome. The SNP's influence on the outcome must be mediated exclusively through its effect on the exposure, ensuring no direct impact on the outcome beyond this pathway.

Our research examined IBD, CD, and UC as exposure variables. We utilized the IIBDGC, which comprises data on 12,882 individuals diagnosed with IBD, including 5956 cases of CD and 6968 cases of UC, all of European descent (Liu et al. 2015). A rigorous control series was implemented to identify eligible IVs. Specifically, IVs for IBD were chosen from SNPs associated with the disease at a genome‐wide significance level (*p *< 5 × 10^−8^) (Zeng et al. 2023). In order to incorporate more SNPs associated with 91 inflammatory proteins, we adjusted the threshold for inflammatory proteins by raising it to *p* < 1 × 10^−5^, allowing more SNPs as IVs. To ensure the independence of the IVs for IBD, we applied a clumping strategy with an *r* ^2^ threshold of < 0.001 and a clump window of 10,000 kb, based on the 1000 Genomes Project linkage disequilibrium (LD) reference panel for European populations (Lu et al. 2023). Applying the standard quality control measures outlined above, 65 SNPs, 53 SNPs, and 39 SNPs were selected as IVs for the exposure. We then calculated the *F*‐statistics for all these SNPs to assess the strength of the genetic instruments, finding that all exhibited *F*‐statistics greater than 10. The IVs utilized in this study are detailed in Tables S1–S3.

### 2.4 Protein–Protein Interaction Network Construction

We constructed the protein–protein interactions (PPI) using STRING (https://string‐db.org/) with a confidence score threshold of 0.4 as the minimum interaction score required, while all other parameters were kept at their default settings. The resulting PPI network was used to visualize potentially meaningful PPI interactions.

### 2.5 Statistical Analysis

To investigate the potential causal relationship between IBD—encompassing CD and UC—BP, we employed several statistical methodologies to identify potential causal relationships. These methods included inverse variance weighted (IVW), MR‐Egger regression, and the weighted median approach. Each technique was utilized to rigorously analyze the data for evidence of causality, thereby ensuring the robustness of our findings. IVW was used as the primary method for MR analysis. MR‐Egger and weighted median were added as sensitivity analysis methods. In addition, we used MR‐PRESSO and leave‐one‐out analyses to assess horizontal multiplicity and identify outliers. Statistical analyses were performed using the TwoSampleMR (version 0.6.1) and MR‐PRESSO (version 1.0) packages in R (version 4.2.3), and plots were created using the ggplot2 package. A *p* < 0.05/3 (with Bonferroni corrections) was considered statistically significant, with *p* values between 0.05 and 0.0167 considered suggestively significant. We interpreted the results not solely based on *p* values but also considered the strengths of the associations and the consistency across sensitivity analyses.

## 3 Results

### 3.1 Causal Effect of IBD and Its Subtypes on BP

**FIGURE 2:**
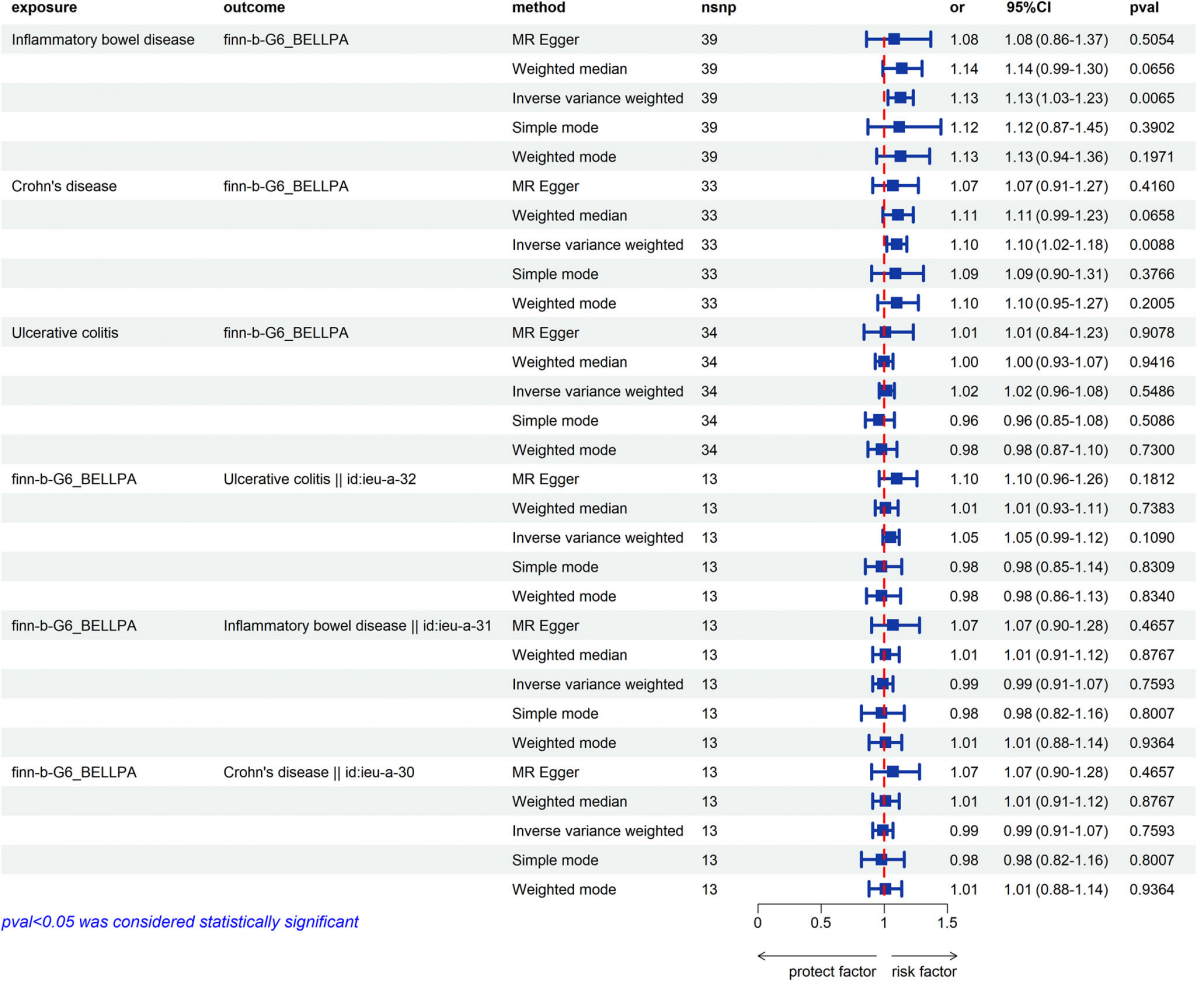
The forest plot of all the results.

Figure 2 clearly illustrates the causal relationship between IBD and BP. This study definitively indicates that IBD, including CD, is a risk factor for BP (Tables S4–S12). The IVW method identified significant causal relationships. Data from the FinnGen consortium demonstrated a significant positive association between IBD and BP (OR: 1.13, 95% CI [1.03–1.23], *p =* 0.0065). CD was also observed to be positively correlated with BP (OR: 1.10, 95% CI [1.02–1.18], *p =* 0.0088 < 0.0167). After applying the Bonferroni correction, IBD remained positively correlated with BP. The heterogeneity test showed no heterogeneity between the included exposure tools (IBD, CD, and UC) and BP. The horizontal pleiotropy test also revealed no horizontal pleiotropy. The scatterplot suggests a positive causal relationship between IBD, CD, and BP (Figure S1). Furthermore, the funnel plot shows no heterogeneity between the three conditions (Figure S2). The leave‐one‐out plot also indicates the stability of this study model (Figure S3).

### 3.2 Exploration of the Causal Relationship Between 91 Inflammatory Proteins and IBD

**FIGURE 3:**
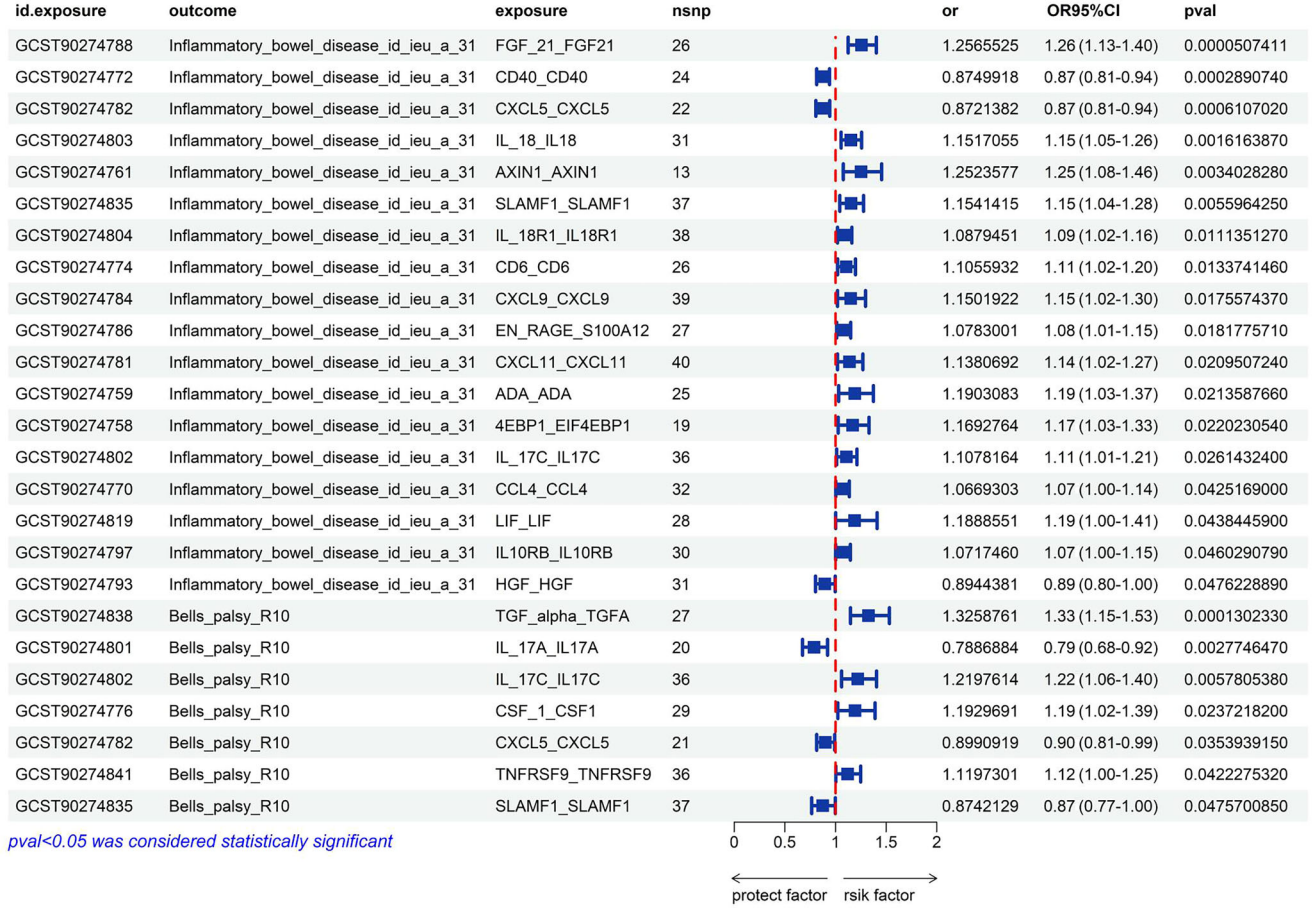
The forest plot of the 91 inflammatory proteins results. CXCL5, IL17_C, and SLAMF1, are inflammatory proteins with common potential causality across IBD and BP.

A causal relationship between 91 inflammatory proteins and IBD from a two‐sample MR study suggests that 18 inflammatory proteins, including FGF21, CD40, C‐X‐C motif chemokine ligand 5 (CXCL5), IL18, IL17C, and signaling lymphocytic activation molecule family member 1 (SLAMF1), are potentially causally associated with IBD. After FDR correction, there was a significant causal relationship between FGF21, CD40, CXCL5, IL18, and IBD, as detailed in Figure 3 (Tables S13 and S14). PPI network indicated that CXCL5, CXCL9, CXCL11, CCL4, CD40, IL18, and HGF were the core of these 18 proteins (Figure S4).

### 3.3 Exploration of the Causal Relationship Between 91 Inflammatory Proteins and BP

A causal relationship between 91 inflammatory proteins and BP from a two‐sample MR study showed that seven inflammatory proteins, including TGFα, IL17A, IL17C, CXCL5, and SLAMF1, were potentially causally related to IBD. After FDR correction, there was a significant causal relationship between TGFα and BP (OR: 1.33, 95% CI [1.15–1.53], pFDR = 0.012 < 0.05), as detailed in Figure 3 (Tables S15 and S16). The PPI network indicated that CSF1 and IL17A were the core of these seven proteins (Figure S5).

### 3.4 Exploring the Relationship Between All Inflammatory Proteins That Have a Potential Relationship With IBD and BP

**FIGURE 4:**
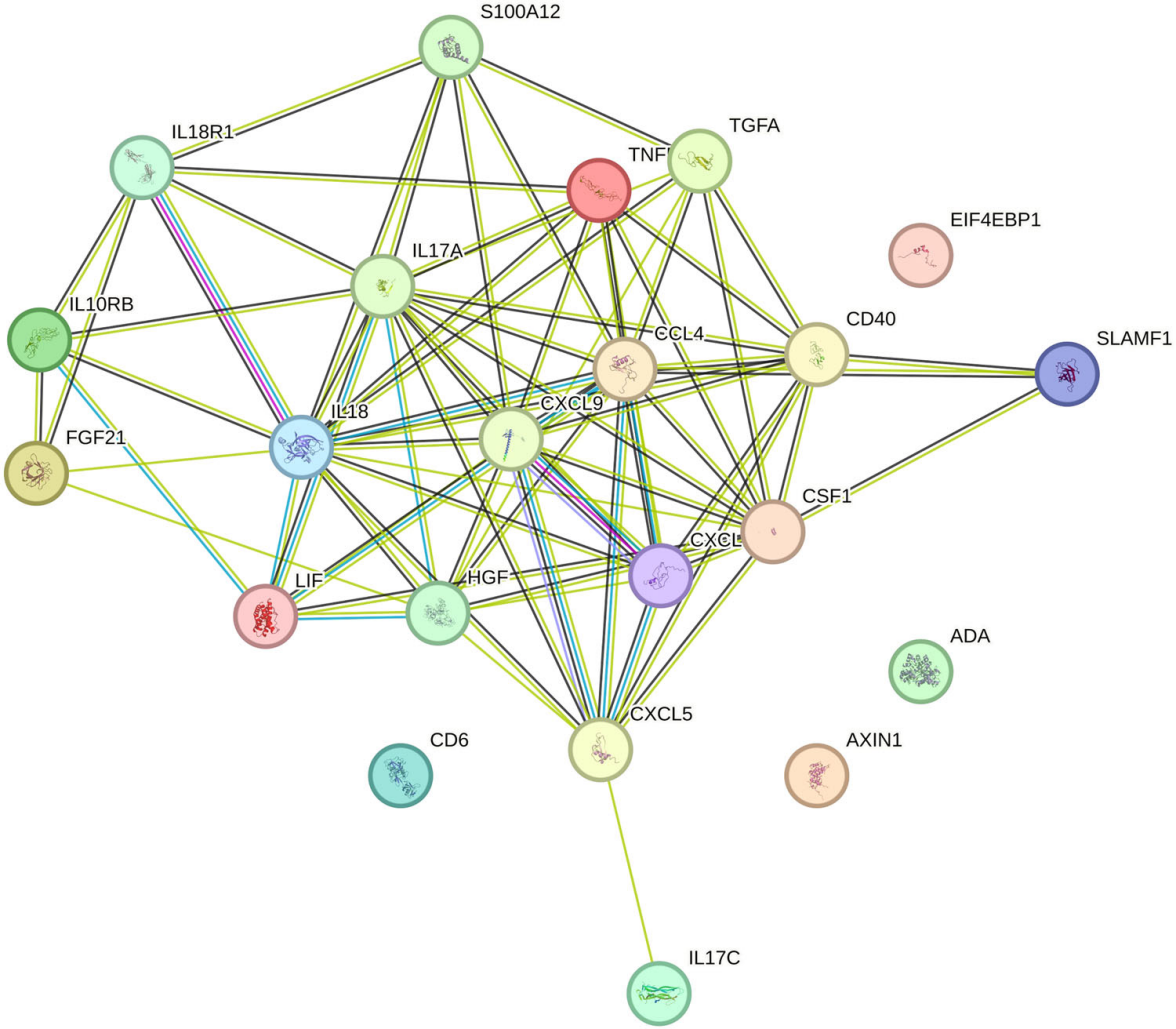
PPI network diagram of inflammatory factors potentially associated with IBD and BP. CXCL5 plays an important role in the regulation of IL_17C and SLAMF1.

There are 23 inflammatory proteins that are potentially causally related to either IBD or BP, with CXCL5 and SLAMF1 potentially causally related to both diseases. We loaded 23 inflammatory proteins into the STRING database to create a PPI network. The results are shown in Figure 4, including a protein interaction pathway consisting of 23 nodes and 42 edges. Among them, CXCL5, CXCL9, CXCL11, CCL4, IL17A, and IL18, as the core of this network, have significant interactions with other inflammatory proteins. CXCL5 and SLAMF1, which are both associated with these two diseases, also interact. However, while CXCL5 is protective against BP and IBD, IL_17C appears to be a risk factor for the two diseases. SLAMF1, on the other hand, is protective for BP and a risk factor for IBD (detailed in Figure 4 and Table S17).

## 4 Discussion

This two‐sample MR study investigates the relationship between IBD and BP using summary statistics from the IIBDGC and BP data from FinnGen R10. These insights could be crucial in managing BP risk factors. The results of this study showed a significant positive causal relationship between IBD and BP. Our findings indicate that IBD, including CD, is a risk factor for BP. This finding is consistent with the higher prevalence of BP among IBD patients observed in previous epidemiologic studies. The results of an MR study of 91 inflammatory proteins across IBD and BP patients suggest that CXCL5 is protective against both, while IL_17C is a risk factor for the two diseases. Alternatively, SLAMF1 is a protective factor for BP and a risk factor for IBD. The finding that genetic variants associated with IBD were also linked to the risk of BP further supports a possible common mechanism of inflammation in both diseases.

Few observational studies have reported an association between IBD and BP, with only a few cases of IBD complicating BP. This two‐sample MR study suggests that genetic predisposition to IBD may contribute to the pathogenesis of BP. Several potential mechanisms could explain this relationship. On the one hand, there are systemic inflammatory mechanisms (Orrù et al. 2020). IBD involves chronic inflammation of the gastrointestinal tract, which can lead to systemic inflammation. This chronic inflammatory state may impact various organs and systems, including the peripheral nervous system. Previous studies have documented that IBD can mediate diseases, such as myasthenia gravis, polymyositis, and dermatomyositis (Ferro and Oliveira Santos 2021). Moreover, systemic inflammation indices have been identified as markers and prognostic indicators for BP (Kınar et al. 2021). The MR study exploring the relationship between 91 inflammatory proteins and IBD suggests interleukin‐18 (IL‐18) as a potential risk factor for IBD. IL‐18 is a pro‐inflammatory cytokine that regulates inflammatory and immune responses. Studies have indicated that IL‐18 mediates neuroinflammation under pathological conditions, which supports the notion that IL‐18, among IBD risk factors, may increase the risk of BP. However, another MR study between 91 inflammatory proteins and BP suggests that CXCL5 and SLAMF1 may be protective against BP.

CXCL5 is an important neutrophil chemokine, and neutrophils act as immunomodulators both to promote inflammatory responses and, in some cases, to protect against them (Balog et al. 2023). CXCL5 serves as a biomarker of prognosis in neurodegenerative diseases, neuropathic pain, peripheral nerve injury diseases, and tumors (W. Zhang, Wang et al. 2020), whereas it is rarely mentioned in BP. The mechanism of BP is widely recognized to involve damage to the facial nerve due to the inflammatory response triggered by HSV infection (Morgan et al. 1995; McCormick 1972; Zimmermann et al. 2019). HSV infection contributes to the massive expression of CXCL5 through Toll‐like receptor 2 signaling (Aravalli et al. 2005).

SLAMF1 is an immunoregulatory molecule located on the surface of immune cells and belongs to the SLAM family. SLAMF1 is expressed in a wide range of immune cells, such as T‐cells, B‐cells, natural killer cells, and macrophages, and plays a key role in regulating the immune response (Dragovich and Mor 2018). It is involved in the regulation of adaptive and innate immune responses by promoting cell–cell interactions and signaling and enhancing the activation and proliferation of immune cells. Studies have shown that SLAMF1 plays an important role in a variety of immune‐related diseases, such as autoimmune diseases, infections, and tumors (Gordiienko et al. 2019). Activation of SLAMF1 inhibits excessive inflammatory responses and protects tissues from damage (Wei et al. 2024). Several studies have suggested that polymorphisms in SLAMF1 may be associated with susceptibility to IBD (P. Zhang et al. 2022). In addition, SLAMF1 may influence the immune environment in the gut by regulating immune cell activity and cell–cell interactions (Monini et al. 2010). Based on the previously published evidence, SLAMF1 is a risk factor for IBD, in line with the findings of the present study. The results of a study on the relationship between cerebrospinal fluid proteomics and BP additionally suggest that SLAMF1 may help to reduce neuroinflammation and protect the integrity of the facial nerve (Masouris et al. 2023), further corroborating the results of this MR study.

This study suggests that IL_17C is a potential risk factor for IBD. The findings of the current study support the previously reported positive correlation between IL_17C levels and IBD (Friedrich et al. 2015), and previous studies indicating that each isoform of IL‐17 increases the risk of IBD (Cai et al. 2023). In addition, IL‐17 is involved in the pathogenesis of many other inflammatory diseases, including neurological disorders. The results of this MR study suggest that IL_17C is also a potential risk factor for BP. Previous studies have shown that IL‐17 contributes to the neuroinflammatory response after neurological injury (Kim and Moalem‐Taylor 2011). Another study indicated that IL_17C is a neurotrophic cytokine (Peng et al. 2017; Iliev et al. 2023). Thus, IL_17C may be involved in the inflammatory response of the facial nerve in BP.

The mechanism by which IBD may increase the risk of BP may be due to the release of inflammatory proteins CXCL5, IL_17C, and SLAMF1, which are induced by IL‐18. IL‐18 induces the expression of CXCL5, which further contributes to the release of IL_17C and enhances the migration and activation of neutrophils. This upregulation of chemokines not only exacerbates local inflammation but may also lead to a systemic inflammatory response, which in turn affects the expression and function of SLAMF1 and modulates the immune cell response. During neuroinflammation, SLAMF1 may inhibit IL‐18 and CXCL5‐mediated hyperinflammatory responses to some extent by regulating immune cell interactions. By regulating immune cell activity, SLAMF1 may help to reduce neuroinflammation and protect facial nerve integrity. In addition, the regulation of SLAMF1 may also result in milder activation of immune cells, thus avoiding excessive damage to nerve tissue and reducing the incidence of BP.

This study is the first to examine the relationship between IBD and BP using MR. Our results show that IBD increases the risk of BP, emphasizing the importance of preventing IBD to avert major neurological disorders. This study also suggests that CXCL5 and SLAMF1 may act as common pathways of action between facial paralysis and IBD etiology, possibly through mechanisms that regulate immune responses, promote tissue repair, and maintain immune homeostasis. Further studies will contribute to a deeper understanding of the specific roles of CXCL5 and SLAMF1 in these two diseases and explore their potential as therapeutic targets. Although the present study provides genetic evidence for a causal relationship between IBD and BP, several limitations remain. First, our study relied on existing genetic and phenotypic data, which may be subject to selection bias and information bias. Second, although we adjusted for multiple potential confounders, there may still be unidentified or unadjusted confounders. In addition, our study sample was primarily derived from a European population, which may limit the generalizability of the results to broader populations.

## 5 Conclusion

Our study provides compelling evidence that IBD may be a risk factor for BP at the genetic level. These findings underscore the importance of considering neurological complications in the management of IBD. CXCL5, IL_17C, and SLAMF1, as possible co‐acting channels between BP and IBD, may provide new potential targets for the treatment of both diseases.

## Author Contributions

**Daofeng Fan**: conceptualization, data curation, formal analysis, software, methodology, writing – original draft, visualization, writing – review and editing. **Wenbao Wu**: conceptualization, data curation, formal analysis, software, methodology, writing – original draft, funding acquisition, investigation, writing – review and editing. **Jiaqian Dai**: validation, software, supervision, resources, visualization. **Yinjuan Chen**: investigation, visualization, formal analysis. **Qingqing Lian**: formal analysis, software, writing – review and editing, visualization. **Changbo Zhao**: data curation, writing – review and editing. **Binfu Que**: data curation, writing – review and editing. **Rui Qiu**: data curation, writing – review and editing, project administration.

## Ethics Statement

This research did not increase the risk or economic burden of patients. The patients’ rights were fully protected. The project design was conducted in line with scientific and ethical principles. All data used in this study were retrieved from previously published GWAS. Each institutional review board in this research obtained written informed consent from all the participants in their respective studies.

## Conflicts of Interest

The authors declare no conflicts of interest.

## Peer Review

The peer review history for this article is available at https://publons.com/publon/10.1002/brb3.70715

## Supporting information




**Supporting Fig.1**: brb370715‐sup‐0001‐Figures.pdf

**Supporting Table1**: brb370715‐sup‐0002‐Table.xls

## Data Availability

The GWAS summary statistics for Bell's palsy are available on the IEU Open GWAS project consortium website (https://gwas.mrcieu.ac.uk/) and the FinnGen R9 website (https://www.finngen.fi/en). The GWAS summary statistics for inflammatory bowel disease are available on the International Inflammatory Bowel Disease Genetics Consortium (https://www.ibdgc.org/). The data generated or analyzed in this study are available in this published article and its Supplementary Information document.
