# Supplementary material for: Exploring the Causal Relationship Between Inflammatory Bowel Disease and Bell's Palsy Based on Inflammatory Proteins: A Mendelian Randomization Study
Source: Brain Behav. 2025 Aug 4;15(8):e70715. doi: 10.1002/brb3.70715 (PMC12321965; doi:10.1002/brb3.70715)

Supplementary Figure 1. The scatterplot of causal relationship between IBD,CD,and Bell's palsy.

MR Test

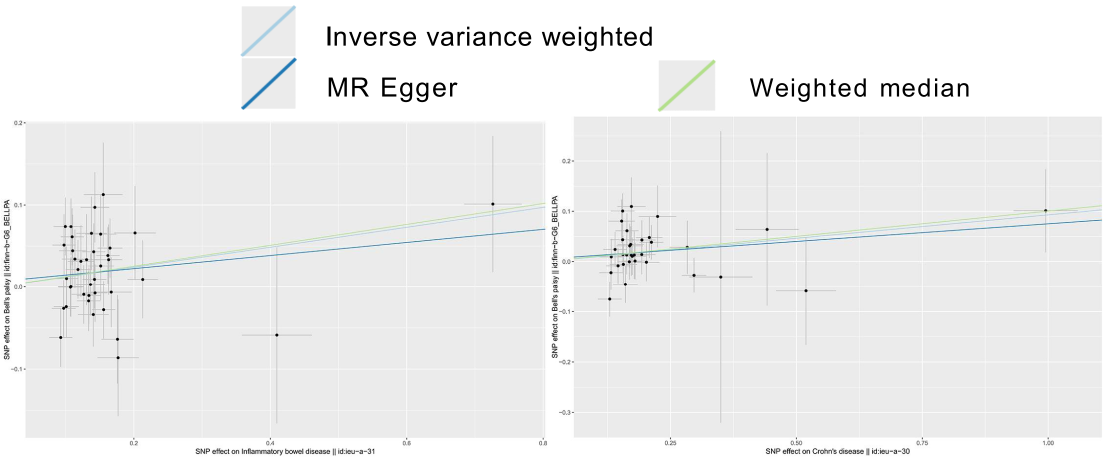

Supplementary Figure 2. The funnel plot of causal relationship between IBD,CD,and Bell's palsy.

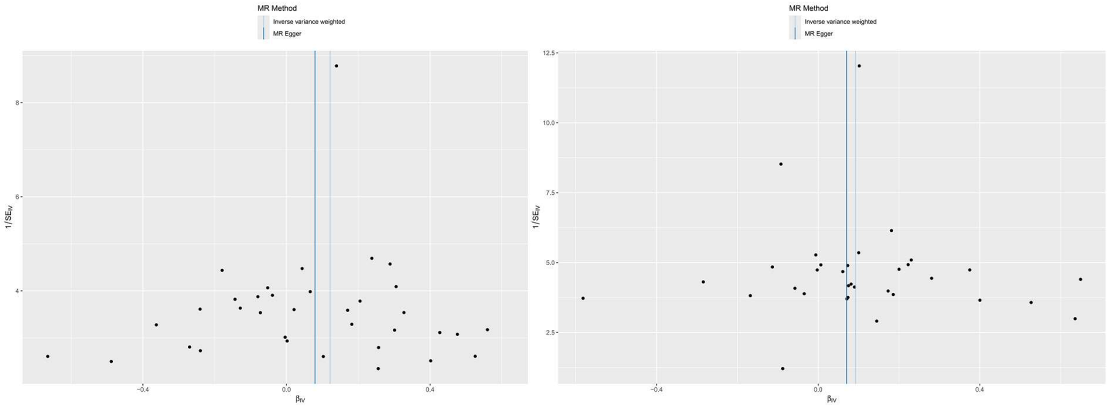

Supplementary Figure 3. The leave-one-out plot of causal relationship between IBD, CD, and Bell's palsy

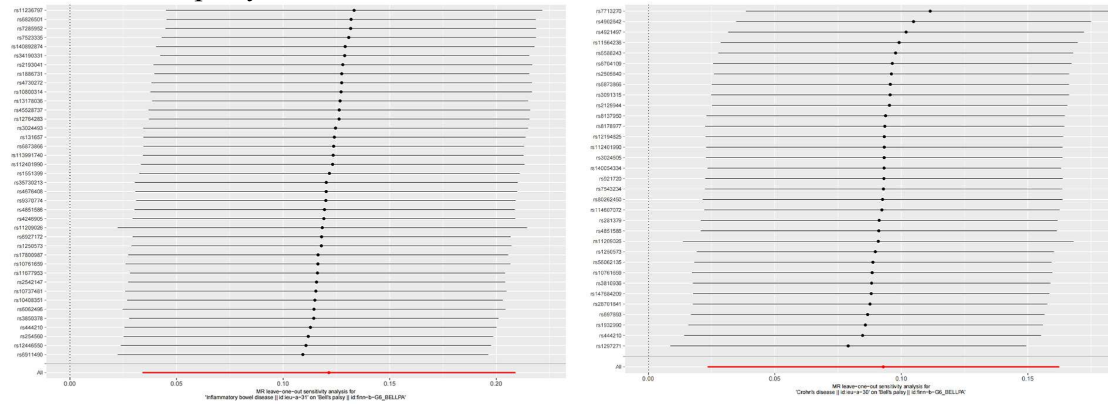

Supplementary Figure4. PPI network diagram of inflammatory factors potentially associated with Bell's palsy

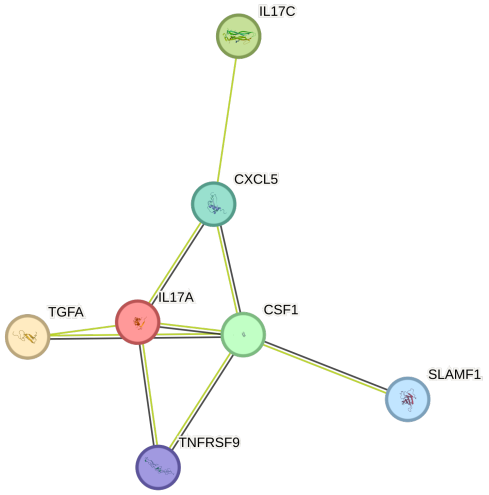

Supplementary Figure5. PPI network diagram of inflammatory factors potentially associated with inflammatory bowel disease

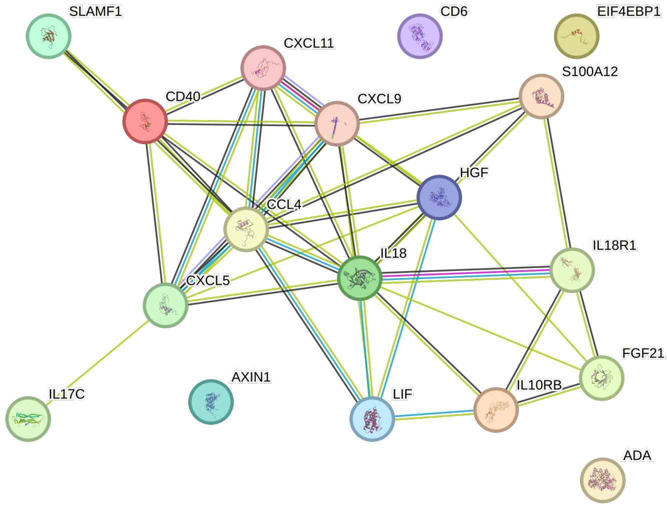

Supplement: Supplementary file 1 — Supporting Fig.1: brb370715‐sup‐0001‐Figures.pdf [file BRB3-15-e70715-s002.pdf]
